# Genomic Strategies in Pediatric Care: Addressing Rare Diseases in Children

**DOI:** 10.3390/children13091194

**Published:** 2026-09-04

**Authors:** Natàlia Caelles-Gramunt, Jordi Pijuan

**Affiliations:** 1 DiNA Science, C/Tuset, 23, 08006 Barcelona, Spain; 2 Applied Research in Neuromuscular Diseases, Institut de Recerca Sant Joan de Déu, C/Santa Rosa, 39-57, Esplugues de Llobregat, 08950 Barcelona, Spain

**Keywords:** rare diseases, genome sequencing, genomic medicine, precision medicine, molecular diagnosis

## Abstract

Background: Rare diseases collectively affect millions of children worldwide and are a major cause of pediatric morbidity, mortality, and lifelong disability. Although most have a genetic basis, obtaining a timely molecular diagnosis remains challenging because of substantial clinical and genetic heterogeneity. Advances in genomic medicine are transforming rare disease diagnosis and establishing genomics as the center of precision medicine. Methods: This review summarizes current evidence on genomic approaches for pediatric rare diseases, including established and emerging sequencing technologies, their clinical applications, implementation challenges, and future directions. Results: Whole-genome sequencing is increasingly being adopted as a first-line genomic test for suspected rare genetic disorders, particularly when the phenotype is heterogeneous or does not point to a specific diagnosis. Conventional cytogenetic and targeted molecular techniques remain important complementary approaches for selected phenotypes, variant classes, and orthogonal confirmation. Gene panels are effective for well-defined phenotypes, whereas whole-exome sequencing remains a high-yield approach for genetically heterogeneous disorders, particularly when whole-genome sequencing is not available or is not clinically indicated. Long-read whole-genome sequencing expands diagnostic capacity by detecting structural variants, repeat expansions, complex rearrangements, and non-coding pathogenic variants that frequently escape short-read technologies. Emerging multi-omics approaches further improve variant interpretation and help resolve previously unsolved cases. Beyond diagnosis, molecular findings guide personalized clinical management, genetic counselling, reproductive planning, and access to targeted therapies and genotype-driven clinical trials. However, broad implementation is constrained by challenges in variant interpretation, ethical and legal considerations, data governance, workforce capacity, cost, and inequitable access to genomic services. Artificial intelligence, international data-sharing initiatives, and coordinated healthcare networks are helping overcome these barriers and improve diagnostic equity. Conclusions: Whole-genome sequencing is increasingly emerging as a first-line genomic strategy for pediatric rare diseases, while complementary technologies, expert phenotyping, and iterative data interpretation remain essential for comprehensive and accurate diagnosis and equitable access to genomic medicine.

## 1. Introduction

Rare diseases (RDs) encompass a highly heterogeneous group of disorders that together impact approximately 3.5–5.9% of the global population. Despite the low individual prevalence of each condition, RDs collectively pose a significant public health challenge [1,2,3,4]. Over 7000 RDs have been identified, approximately 70–80% of which have an underlying genetic cause, and the majority manifest during childhood [4,5]. As a result, RDs exert a disproportionate impact on child health, contributing notably to infant mortality, pediatric hospitalizations, and chronic disability. While rare individually, these disorders are frequently chronic, debilitating, and life-limiting, collectively imposing substantial medical, psychosocial, and socioeconomic burden on patients, families, and healthcare systems [5,6].

Despite remarkable advances in the genetics area, obtaining a definitive diagnosis remains one of the greatest challenges for children with suspected rare genetic diseases [7,8]. Marked genetic and phenotypic heterogeneity, incomplete knowledge of disease mechanisms, and unequal access to genomic testing contribute to a prolonged diagnostic odyssey, often lasting years and involving multiple specialist consultations, repeated investigations, and inconclusive results. Even after traditional genetic techniques and exome sequencing, a substantial proportion of patients remain undiagnosed, highlighting the need for more comprehensive genomic approaches, periodic reanalysis of genomic data, improved variant interpretation, the integration of complementary multi-omics technologies and functional studies [5,9,10]. Recent studies have demonstrated that whole-genome sequencing (WGS), long-read sequencing (LRS), advanced bioinformatic pipelines, and systematic reinterpretation of genomic data continue to improve diagnostic yield in previously unsolved cases [9].

Obtaining a genetic diagnosis has become a cornerstone of modern RD care. Beyond ending the diagnostic odyssey, a confirmed genetic diagnosis improves prognostic accuracy, guides disease-specific management and surveillance, informs recurrence risk and reproductive counselling, and connects families with disease-specific support networks. Importantly, it also identifies patients who may benefit from targeted therapies, enables enrollment in clinical trials, and facilitates access to an expanding landscape of precision medicine interventions [4]. Growing evidence demonstrates that genomic diagnosis changes clinical management for a substantial proportion of patients, reinforcing genome-scale sequencing as a first-line diagnostic approach for individuals with suspected monogenic disorders.

**Figure 1:**
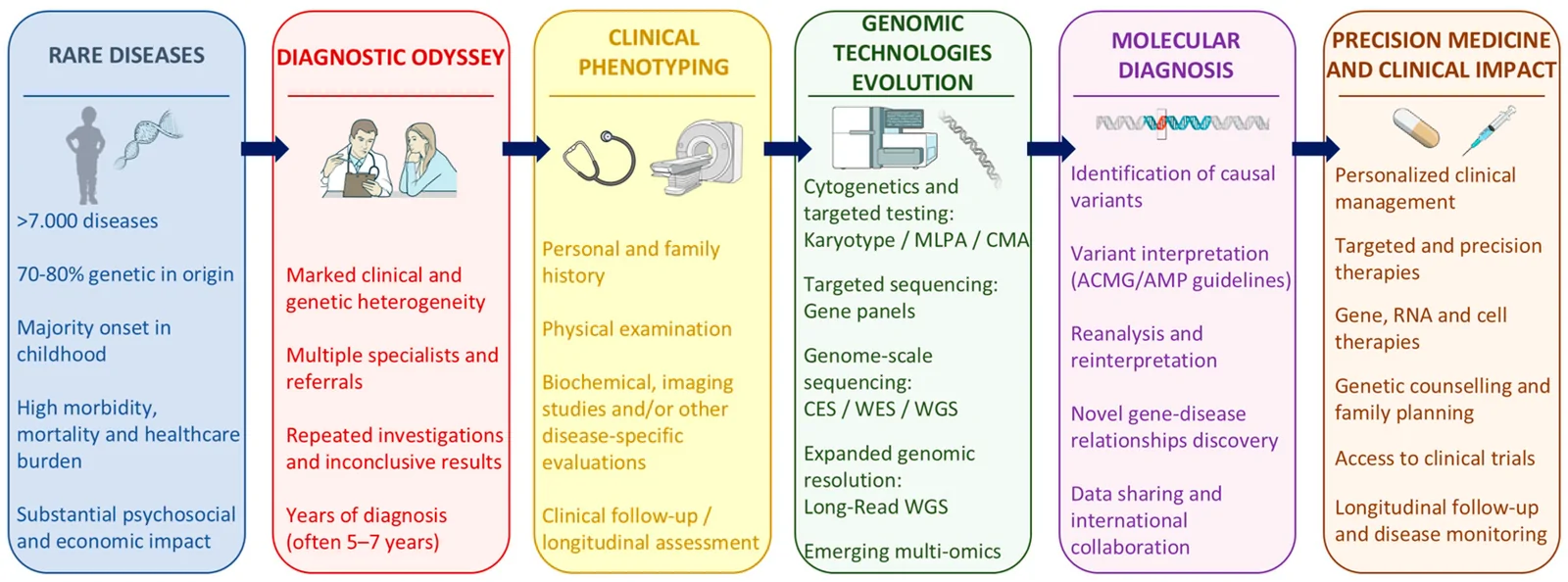
The evolving role of genomics in RD diagnosis. RD often involve prolonged diagnostic odysseys due to their marked clinical and genetic heterogeneity. Genome-scale sequencing, together with emerging multi-omics approaches, is transforming molecular diagnosis and enabling precision medicine through personalized management, genetic counselling, and access to targeted therapies and clinical trials. The figure was, in part, created with NIH BioArt source.

The widespread adoption of genome-scale sequencing (short-read and long-read sequencing) has revolutionized RDs diagnosis, shifting from a gene-by-gene approach to a comprehensive genomic strategy. This has established genomics as a center pillar of precision medicine and personalized care (Figure 1) [11,12]. In addition to improving diagnostic yield, genome sequencing enables detection of a wider range of pathogenic variants—including structural variants (SV), repeat expansions, deep intronic variants, mitochondrial variants, and complex genomic rearrangements—while also accelerating the discovery of novel disease genes through data sharing and international collaboration. As the number of targeted therapies, gene therapies, RNA-based treatments, and mechanism-driven clinical trials continues to expand, timely molecular diagnosis is increasingly the gateway to precision therapeutics. Therefore, equitable access to comprehensive genomic testing has become an essential component of modern healthcare systems, ensuring that individuals with RDs can benefit from emerging therapeutic opportunities and participate in clinical research.

In this review, we examine how genomic medicine is reshaping the diagnosis and clinical management of pediatric RDs, from the diagnostic odyssey to precision medicine. We evaluate the evolution of genomic technologies—from conventional cytogenetic approaches to genome-scale sequencing, particularly long-read whole-genome sequencing, and emerging multi-omics strategies—and discuss their impact on molecular diagnosis, personalized care, genetic counselling, and access to targeted therapies and genotype-driven clinical trials. We further address the scientific, ethical, and implementation challenges that continue to limit the widespread adoption of genomic medicine, and explore future directions, including artificial intelligence (AI), international collaborative initiatives, and sustainable integration into healthcare systems, with the ultimate goal of improving diagnostic equity and enabling precision medicine for all children with RD.

## 2. Genomic Technologies

**Figure 2:**
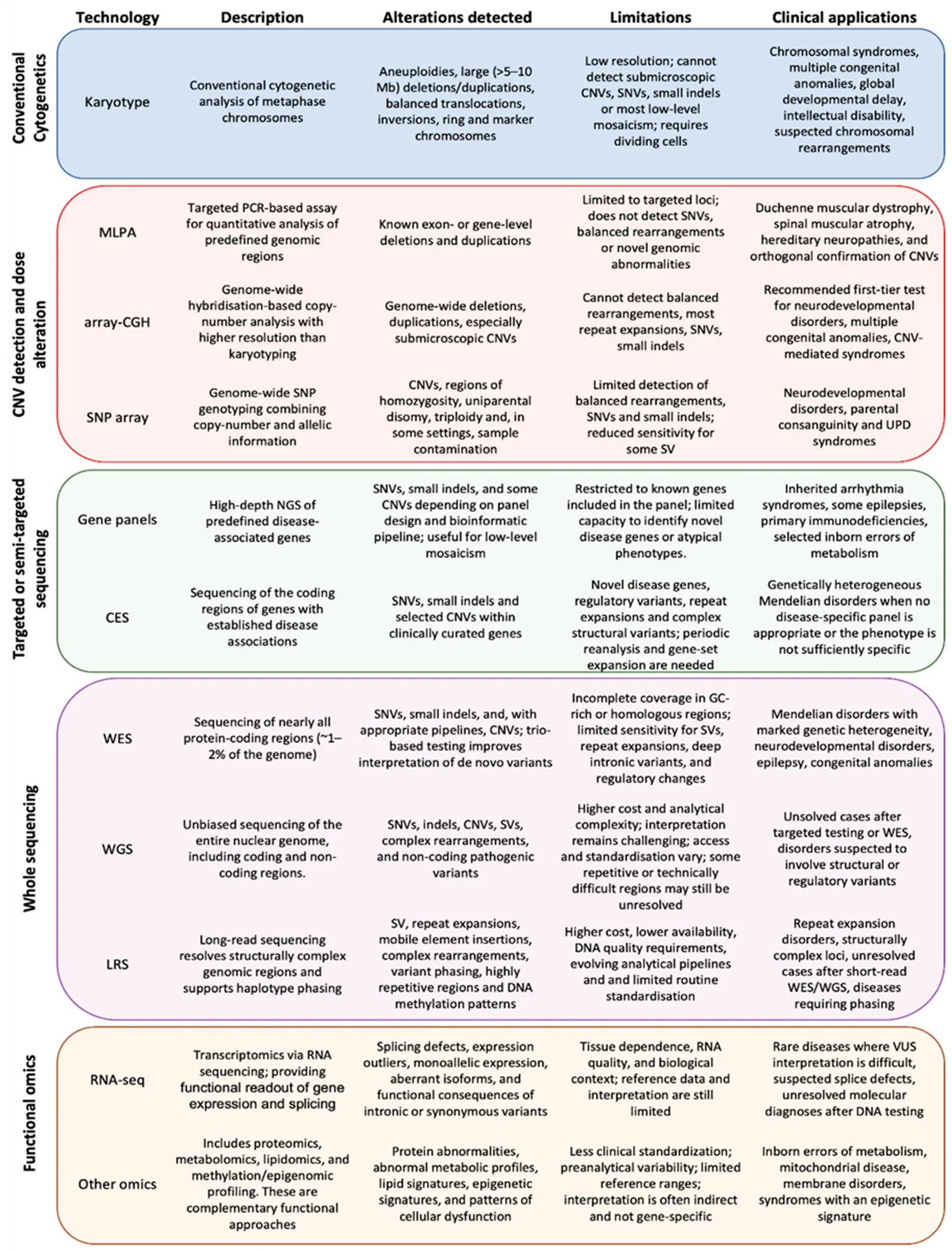
Summary of genomic technologies used in the diagnosis of pediatric RD. The table compares the analytical scope, variant classes detected, major limitations and preferred clinical applications of conventional cytogenetic, molecular and sequencing-based technologies. Functional omics approaches are included as complementary tools to facilitate the interpretation of genomic variants and increase the diagnostic yield of unresolved cases.

Next-generation sequencing (NGS) has transformed the diagnostic approach to rare pediatric disorders, with WGS increasingly moving toward first-line use in clinical practice. Rather than representing a linear sequence of tests, current genomic diagnostics comprise a set of complementary approaches whose selection depends on the clinical phenotype, the suspected variant class, the urgency of diagnosis, and the capabilities of the testing platform. WGS can consolidate the detection of multiple variant classes within a single assay, but targeted and cytogenetic techniques remain valuable when a specific genomic mechanism is suspected, when particular variant classes require orthogonal testing or confirmation, or when WGS does not yet provide sufficient analytical sensitivity [5,13]. In the context of RDs, genomic and omics technologies should be regarded as a set of partially overlapping techniques that complement one another and are selected according to the expected variant type, the precision of the phenotypic definition, and the clinical purpose of the test (Figure 2). Indeed, many cases of misdiagnosis arise from a mismatch between the phenotype, the variant type, and the design of the analysis, rather than from a lack of genomic information [13].

Genomic testing should be preceded by a structured clinical assessment rather than viewed as an isolated laboratory investigation. Phenotyping includes a detailed personal and family history, review of the clinical course, physical examination—including growth, neurodevelopmental and dysmorphological assessment when appropriate—and targeted non-genetic investigations such as basic laboratory and biochemical testing, imaging, and other disease-specific evaluations. This clinical information is essential for selecting the most appropriate genomic strategy and for subsequent interpretation of sequencing results, including prioritization of candidate genes and assessment of genotype–phenotype concordance [14]. Systematic collection of clinical, biochemical, morphological, and imaging features can further strengthen phenotype-based gene prioritization and facilitate the interpretation and reanalysis of genomic data. Standardized phenotypic frameworks, such as the Human Phenotype Ontology (HPO), can facilitate structured communication and computational phenotype matching, but should complement rather than replace expert clinical assessment.

### 2.1. First-Line Genome Sequencing and Complementary Genomic Technologies

WGS is increasingly being implemented as a first-line genomic test for children with suspected rare genetic disorders, particularly when the phenotype is heterogeneous, evolving, or does not point to a specific molecular diagnosis. Evidence from recent studies supports its use as an initial strategy in selected pediatric settings, with diagnostic yields generally around 30–40% and higher yields reported in some first-line cohorts [15,16]. This transition is already evident in some national healthcare systems. Denmark, for example, has implemented WGS as a first-tier diagnostic approach for suspected monogenic disorders within the Danish National Genome Centre, with recent population-scale experience demonstrating the feasibility of integrating WGS into routine clinical diagnostics [17].

However, the incremental diagnostic yield of WGS over WES is generally modest, and no single genomic technology captures all clinically relevant variant classes with equal sensitivity (Table 1). The role of WGS should therefore be understood within a broader diagnostic framework in which targeted and cytogenetic methods remain complementary tools for specific phenotypes, variant types, confirmation of genomic findings, or circumstances in which WGS has recognized limitations.

Although increasingly superseded by genome-scale sequencing for many indications, conventional cytogenetics remains valuable (Table 1). Karyotyping remains useful when large chromosomal abnormalities or balanced rearrangements are specifically suspected and when a global assessment of chromosome number and structure is clinically informative [16,18]. Chromosomal microarray analysis (CMA), including array comparative genomic hybridization and single nucleotide polymorphism (SNP) arrays, remains useful for detecting genome-wide copy-number abnormalities, including submicroscopic deletions and duplications [19] and regions of homozygosity [20]. Its role is increasingly selective, including settings where WGS is unavailable, where a specific genomic mechanism is suspected, or where array-based analysis provides complementary information. As WGS increasingly incorporates robust CNV and structural-variant detection, CMA is being replaced as a first-line investigation in some healthcare systems. Multiplex ligation-dependent probe amplification (MLPA) remains an important targeted method for detecting exon-level copy-number changes in predefined genes, particularly when a specific disorder or genomic mechanism is suspected or when an orthogonal confirmation of a sequencing finding is required [16,18]. Its relatively low cost, rapid turnaround and high analytical sensitivity for targeted exon-level dosage changes continue to support its use alongside genome-wide sequencing, including for genes or variant classes that may not be reliably resolved or confirmed by exome-based approaches (Table 1).

**Table 1:** Representative diagnostic yields and incremental diagnostic value of genomic technologies in pediatric rare diseases.

Technology	Representative Diagnostic Yield	Incremental Diagnostic Yield	Main Contribution
**Karyotype**	Variable; indication-dependent	—	Aneuploidy, large/balanced chromosomal abnormalities
**CMA**	~10–20% ^1,2^	—	Genome-wide CNVs
**MLPA**	Variable; indication-dependent	Phenotype/gene-dependent	Targeted exon-level CNV detection and confirmation
**Gene panels**	~20–40% ^3,4^	Phenotype-dependent	High-depth targeted sequencing
**WES**	~25–40% ^5,6^	Reference genomic approach	Coding SNVs/indels
**WGS**	~30–40% ^5,7^	Modest incremental yield over WES; ~5–10% in selected comparative cohorts	Coding + non-coding + CNV/SV
**Long-read WGS**	Emerging; highly cohort-dependent	Particularly useful for unresolved cases	Repeat expansions, SVs, phasing, repetitive regions
**Reanalysis**	—	~5–15% additional diagnoses, depending on interval and cohort ^8^	New genes, variant reinterpretation, phenotype evolution
**RNA-seq/multi-omics**	Variable depending on the omics technique	Often case-dependent	Functional resolution of unresolved variants

^1^ [21], ^2^ [22], ^3^ [23], ^4^ [24], ^5^ [25], ^6^ [26], ^7^ [16], ^8^ [27]. Diagnostic yields are representative ranges derived from published cohorts and should not be interpreted as directly comparable across technologies. Yield varies substantially according to clinical indication, phenotype, cohort composition, family structure, prior testing, analytical pipelines, variant classes assessed, and the definition of a molecular diagnosis. Incremental diagnostic yield refers to the additional diagnoses attributable to a technology or analytical strategy compared with the preceding or comparator approach.

### 2.2. Gene Panel

The gradual incorporation of NGS into routine clinical practice has shifted the diagnostic balance between hypothesis-driven and hypothesis-free testing [5]. In pediatric genetics, gene panels and exome-based testing therefore remain clinically important complementary approaches, rather than interchangeable alternatives, with their relative value determined by phenotype specificity and the maturity of gene–disease knowledge [28].

When the clinical presentation is well defined and the relevant genes are already established, targeted panels are often the most efficient option (Table 1). Their narrow scope allows high sequencing depth, which improves sensitivity for mosaic variants and small insertions and deletions, while also reducing the burden of variants of uncertain significance (VUS) and simplifying interpretation [5]. Panels also limit incidental findings, which is particularly relevant in pediatrics, where analysis is generally restricted to genes directly pertinent to the indication [28]. Accordingly, panels are well suited to disorders with relatively stable gene architecture, such as inherited arrhythmia syndromes, some epilepsies and selected inborn errors of immunity [29].

Their principal limitation is conceptual rather than technical: they depend on prior knowledge of the causal gene set. In disorders in which gene discovery remains active or phenotype–genotype boundaries are incomplete, narrowly targeted testing can miss the relevant gene altogether, even when sequencing quality is high. Periodic panel redesign can partially mitigate this problem, but it cannot substitute for the broader search space of exome-based approaches [28].

### 2.3. Clinical Exome Sequencing

Clinical exome sequencing (CES) targets the coding regions of a predefined set of genes with established or putatively relevant Mendelian disease associations, thereby reducing the analytical and interpretative burden relative to unfiltered whole-exome sequencing (WES) and facilitating more standardized laboratory workflows and shorter turnaround times [25,30]. Its diagnostic value rests on the observation that many clinically interpretable variants occur in genes with a strong gene–disease relationship, so restricting analysis to genes with recognized clinical relevance can increase efficiency and limit uncertainty introduced by poorly validated candidate genes (Table 1) [13,28]. Accordingly, CES is often used as a first sequencing strategy for genetically heterogeneous pediatric disorders, particularly when targeted panels are unavailable, too narrow for the phenotype, or insufficiently comprehensive [13,30]. However, its performance depends on rigorous gene selection, adequate coverage, and the capacity for periodic reanalysis and expansion of the analyzed gene set as new gene–disease relationships emerge [28].

### 2.4. Whole-Exome Sequencing, Whole-Genome Sequencing and Long-Read Sequencing

In RD diagnosis, broader genomic strategies are increasingly required when predefined candidate genes do not capture the relevant pathogenic spectrum. This need is particularly evident in pediatrics, where genetic heterogeneity, pleiotropy and phenotype evolution can limit the yield of hypothesis-driven testing [30]. Accordingly, WES, WGS and, more recently, LRS have become key components of precision diagnosis, especially after targeted testing is non-diagnostic. Diagnostic yields in pediatric cohorts are substantial but remain variable, reflecting differences in phenotype, ascertainment and study design (Table 1) [31].

WES remains a practical first-line genomic test because it efficiently interrogates protein-coding regions, where many known pathogenic variants reside. Although coding sequence represents only 1–2% of the genome, its diagnostic yield is favorable relative to sequencing cost and analytical burden, which has established WES as a reference test for genetically heterogeneous Mendelian disorders [31]. Its yield is typically higher than that of targeted panels in broad monogenic disease settings, in part because analysis is not constrained to a fixed gene list (Table 1). With appropriate bioinformatic pipelines, WES can also support copy number variation (CNV) detection, and trio-based testing improves the interpretation of de novo variation and accelerates variant prioritization [32,33].

However, WES is intrinsically limited by its capture-based focus on coding sequence. Coverage can be incomplete in GC-rich or homologous regions, and sensitivity remains limited for SV, repeat expansions, deep intronic variants, regulatory changes and complex rearrangements. As a result, a proportion of unsolved RD cases reflects the technical constraints of short-read exome sequencing rather than the absence of a genetic cause [34].

WGS addresses several of these limitations by extending analysis beyond the exome, improving coverage uniformity and enabling more comprehensive detection of CNV, structural rearrangements and non-coding pathogenic variants. In unresolved patients, WGS can increase diagnostic yield relative to WES, largely by identifying variant classes that exome sequencing does not systematically capture, including cryptic SV, non-coding splice-altering variants and complex genomic rearrangements [35].

Importantly, diagnostic yield is not static. Reanalysis of previously unresolved cases can identify additional diagnoses through new gene disease discoveries, improved filtering, refined variant interpretation, better phenotypic annotation and newly acquired clinical information [36,37]. For this reason, reanalysis is increasingly regarded as part of the diagnostic process rather than an optional add-on (Table 1) [27,37].

WES and WGS are not one-off diagnostic tests, but datasets whose value increases as knowledge accumulates. This is especially relevant in pediatrics, where the phenotype often expands or stabilizes only after the initial diagnostic consultation, altering the interpretative landscape around the same genomic data [28].

Nonetheless, variant interpretation remains a central bottleneck across sequencing platforms. Even when pathogenic variants are detected with relative ease, assigning clinical relevance requires integration of inheritance, allele frequency, predicted impact, functional evidence and genotype–phenotype concordance. This is particularly challenging for missense variants, incomplete penetrance, variable expressivity and multi-locus contributions, underscoring the need for detailed clinical phenotyping alongside molecular analysis [13,28].

Consequently, clinical genomics has evolved from an approach focused exclusively on increasing sequencing yield to one aimed at improving the biological interpretability of genomic variation. This transition has driven the progressive consolidation of exome and genome sequencing in diagnostic practice, as well as the development of long-read technologies capable of resolving structurally complex regions and the incorporation of complementary functional omics to link variant detection with the pathogenic mechanism [31,38].

Long-read sequencing is particularly valuable for haplotype resolution, repeat expansions, structural variation, phasing of compound heterozygous variants and interrogation of genomic regions that remain difficult for short-read methods [13,39,40]. The technology is also gaining importance for disentangling isoform complexity and clarifying structural mechanisms that short-read data may suggest, but cannot resolve [28,40,41,42].

Although the routine implementation of long-read whole-genome sequencing (lrWGS) is currently limited by higher costs, lower throughput, DNA quality requirements, platform-specific error profiles, and the need for standardized analytical pipelines, these barriers are rapidly diminishing as sequencing technologies mature and become more widely adopted [28,41,42]. At present, lrWGS is particularly valuable for resolving cases that remain unsolved after conventional genomic testing, owing to its superior ability to detect SV, repeat expansions, complex genomic rearrangements, and variants within repetitive or poorly accessible genomic regions. As costs continue to decline and analytical performance improves, lrWGS is expected to transition from a complementary problem-solving tool to a first-line genomic test for individuals with suspected rare genetic diseases, providing a single comprehensive assay capable of capturing the full spectrum of clinically relevant genomic variation.

### 2.5. New Emerging Technologies: Transcriptomics, Proteomics, Metabolomics, Lipidomics

Beyond DNA, transcriptomics has become the most established functional complement to genomic testing. RNA sequencing (RNA-seq) enables the detection of splicing alterations, expression changes, monoallelic expression and aberrant isoforms that may go unnoticed or remain ambiguous at the DNA level, making it especially useful in the interpretation of VUS, synonymous variants, intronic variants or variants near splice sites [43,44]. In RDs, RNA-seq is increasingly being incorporated as a complementary tool to support variant interpretation and provide functional evidence of causality [45,46,47]. DNA methylation profiling has also emerged as a useful adjunct in selected disorders with characteristic epigenetic signatures. Genome-wide methylation patterns can function as disease-specific episignatures, providing functional evidence for the pathogenicity of variants and helping distinguish clinically overlapping syndromes. Established examples include disorders associated with chromatin and epigenetic dysregulation, such as Kabuki syndrome, Coffin–Siris syndrome, Rubinstein–Taybi syndrome, and Wiedemann–Steiner syndrome. Episignature analysis can therefore support the interpretation of variants of uncertain significance and, in selected cases, identify a molecular diagnosis that remains unresolved by conventional sequencing [14,48,49].

Proteomics, metabolomics and lipidomics are at an earlier stage of clinical application, but can be informative in metabolic, mitochondrial and membrane disorders, where downstream molecular consequences may be more readily detectable than the causal DNA change itself. Proteomics can provide valuable diagnostic and mechanistic information in selected RDs, although its clinical implementation is still more limited than that of transcriptomics. In addition, these approaches complement genomics and transcriptomics, as the correlation between mRNA and protein levels is not always linear [50,51].

These approaches remain complementary rather than interchangeable, given limitations in reference ranges, pre-analytical variability and clinical interpretation [43,52]. The most effective workflow in children with RD is therefore stepwise: broad genomic testing first, followed by targeted transcriptomic or biochemical studies when DNA analysis is unresolved, and systematic reanalysis as knowledge expands [13,53].

## 3. Clinical Application

Genomics has evolved from a diagnostic tool into a central component of precision medicine in rare pediatric diseases. The clinical value of genomic data lies not only in establishing a molecular etiology, but also in its capacity to reconfigure clinical decision-making by linking pathogenic mechanisms with therapeutic strategies, surveillance programs and recurrence risk assessment. This progress has been driven by the integration of genomic data with detailed clinical phenotyping and functional evidence, which facilitates the identification of intervention opportunities [54,55]. Beyond diagnostic testing in symptomatic children, genomic approaches are also being evaluated as a potential component of newborn screening programs, with the aim of identifying actionable genetic conditions before clinical presentation. Emerging evidence suggests that genomic sequencing can complement or extend conventional newborn screening by enabling the detection of a broader range of severe and potentially treatable genetic disorders, including conditions that may not be captured by current biochemical screening programs [56,57]. However, population-level implementation will require careful evaluation of clinical utility, cost-effectiveness, ethical considerations, and the infrastructure needed for confirmatory testing and long-term follow-up [58].

In pediatric practice, where disease trajectories and therapeutic opportunities can change rapidly, genomic testing is best understood as part of an iterative diagnostic and management process rather than a one-time test result. Reinterpretation of variants over time, integration of longitudinal clinical data and continued contextualization of genomic findings can convert a molecular diagnosis into actionable guidance for treatment, monitoring and recurrence-risk counselling, while also refining the assessment of diagnostic utility beyond a binary yield metric [59].

### 3.1. Personalized Treatment

Genomics has transformed the conceptual framework for rare pediatric diseases by enabling definition of underlying biological mechanisms and shifting classification from organ-based or syndromic labels toward pathway-specific entities [5]. In a subset of disorders, this mechanistic resolution has facilitated selection of targeted interventions and avoidance of ineffective or potentially harmful treatments, although for most RDs the immediate impact remains on clinical management rather than disease-modifying therapy [5,54]. Genomic diagnoses thus serve not only as endpoints but as entry points into mechanism-informed care pathways, including specialist referral, surveillance protocols and eligibility for natural history studies and clinical trials [54].

The relationship between genomic diagnosis and treatment is inherently non-linear. A molecular finding may directly implicate an established therapeutic target, but more commonly it confers clinical utility by clarifying disease mechanism, refining prognosis and enabling access to targeted management strategies [5,54]. The therapeutic relevance of a variant therefore depends not only on its pathogenicity but also on its molecular consequences, tissue specificity, developmental timing and potential reversibility [5]. Even within the same gene, loss-of-function, gain-of-function and dominant-negative variants can necessitate fundamentally different therapeutic approaches, underscoring the need for interpretation at the level of variant and mechanism rather than gene alone [5,54].

This paradigm is especially relevant in pediatrics, where early identification of the molecular defect can enable presymptomatic or early interventions that alter disease trajectory before irreversible damage occurs [54]. Integration of multi-omics approaches has improved discrimination of clinically overlapping disorders and increased the proportion of patients with findings that are potentially ‘clinically actionable’, although the nature of this actionability is often limited to management changes, surveillance or trial eligibility rather than approved targeted therapies [54,55]. For most RDs, a genomic diagnosis does not directly imply therapeutic actionability, and clinical interpretation is further complicated by incomplete penetrance, variable expressivity and genotype–phenotype discordance. In practice, the most consistent impact of genomics is seen in optimization of clinical management, including risk stratification, avoidance of contraindicated interventions and implementation of syndrome-specific surveillance programs [5,54].

Taken together, personalized treatment in rare pediatric diseases should be understood not as a deterministic, one-time decision but as a probabilistic, dynamic and context-dependent framework anchored in the best available mechanistic evidence [5,54]. Genomic and multi-omic data provide the foundation for this framework, but their clinical value is realized only through iterative reinterpretation, integration with detailed phenotyping and alignment with evolving therapeutic and management options [13,54].

### 3.2. Family Impact

The impact of genomic diagnosis on the family should be regarded as an integral component of clinical utility, rather than a secondary benefit [54]. In rare pediatric diseases, the child’s diagnosis is often a family diagnosis: it can explain previous reproductive losses, clarify recurrence risks, identify carrier states and, in some cases, uncover parental mosaicism underlying apparent de novo variants in the proband. Trio-based testing may also reveal unexpected biological relationships, including non-paternity, non-maternity or other forms of non-parentage, as well as previously unrecognized consanguinity; these possibilities should be considered during pre-test counselling because the resulting information may have substantial implications for the child and family. Mitochondrial DNA testing has additional familial implications because pathogenic variants and heteroplasmy can reveal information relevant not only to the child but also to the maternal lineage, potentially affecting interpretation of disease risk in the mother, maternal siblings, and more distant maternal relatives [60]. This information may alleviate uncertainty and guilt, but it can also generate distress, particularly when it revises expectations about future pregnancies or reveals risks of adult-onset disease in parents and siblings [54].

A molecular diagnosis can end a prolonged diagnostic odyssey, provide an explanatory framework for previously unexplained symptoms and reduce uncertainty about the child’s trajectory [59]. These benefits coexist with emotional complexity: parents frequently report relief alongside grief, anxiety and concern about disease progression, and the identification of an inherited variant can reshape perceptions of parental responsibility [59]. Conversely, a de novo finding may reduce concerns about direct transmission but raise questions about recurrence risk in the context of possible parental germline mosaicism [54].

Genetic counselling and cascade testing enable identification of affected or at-risk relatives, facilitate early surveillance and clarify intergenerational recurrence risks, thereby extending the clinical utility of the index diagnosis [61]. However, this familial orientation complicates conventional models of individual autonomy and confidentiality, especially in pediatrics, where parents provide consent but the child’s future autonomy and right to an open future must be considered [61]. These tensions are amplified when results have implications for adult-onset conditions in relatives or when secondary findings are disclosed, requiring careful balancing of beneficence, privacy and the developing autonomy of the child [61].

### 3.3. Genetic Counselling

Genetic counselling is indispensable to the clinical utility of genetic diagnosis, particularly in pediatric genomics [54]. Its functions extend from supporting informed decisions about whether to proceed with testing to helping families interpret results in the context of incomplete penetrance, VUS and secondary findings. In rare pediatric diseases, effective counselling must be proactive, iterative and adapted to the child’s developmental stage, parental expectations and cultural context [54].

Pre-test counselling should address the possibility of uncertain, unexpected or familial findings, including secondary findings, unexpected parentage, consanguinity and, when mitochondrial DNA is analyzed, implications for the maternal lineage. Counselling should also explain the implications of trio sequencing for clarifying inheritance and refining recurrence-risk estimates [62]. Post-test counselling should translate molecular findings into practical decisions about surveillance, treatment and family planning without overstating certainty, while explicitly acknowledging residual uncertainty and the potential for future reinterpretation [54]. This role becomes increasingly critical with the integration of genome sequencing, transcriptomics and other multi-omics approaches, which expand the volume, complexity and longitudinal nature of genomic information [63].

Access to genetic counselling remains unequal, concentrated in centers with specialized infrastructure and limited in many regions and underserved populations. At the same time, the rapid pace of discovery ensures that variant classifications are inherently provisional, with a substantial proportion of variants subject to reclassification over time and heterogeneous practices for reinterpretation and recontact [55]. Genetic counselling is therefore essential for translating genomic information into clinically and ethically meaningful care, providing a structured, longitudinal process through which families can understand uncertainty, weigh consequences and make decisions aligned with their values and circumstances [54]. Realizing this potential will require sustained investment in counselling workforce, standardized approaches to VUS management and recontact, and equitable models of service delivery that extend beyond specialized centers [55].

## 4. Challenges in Implementing Genomic Medicine for Rare Diseases

**Figure 3:**
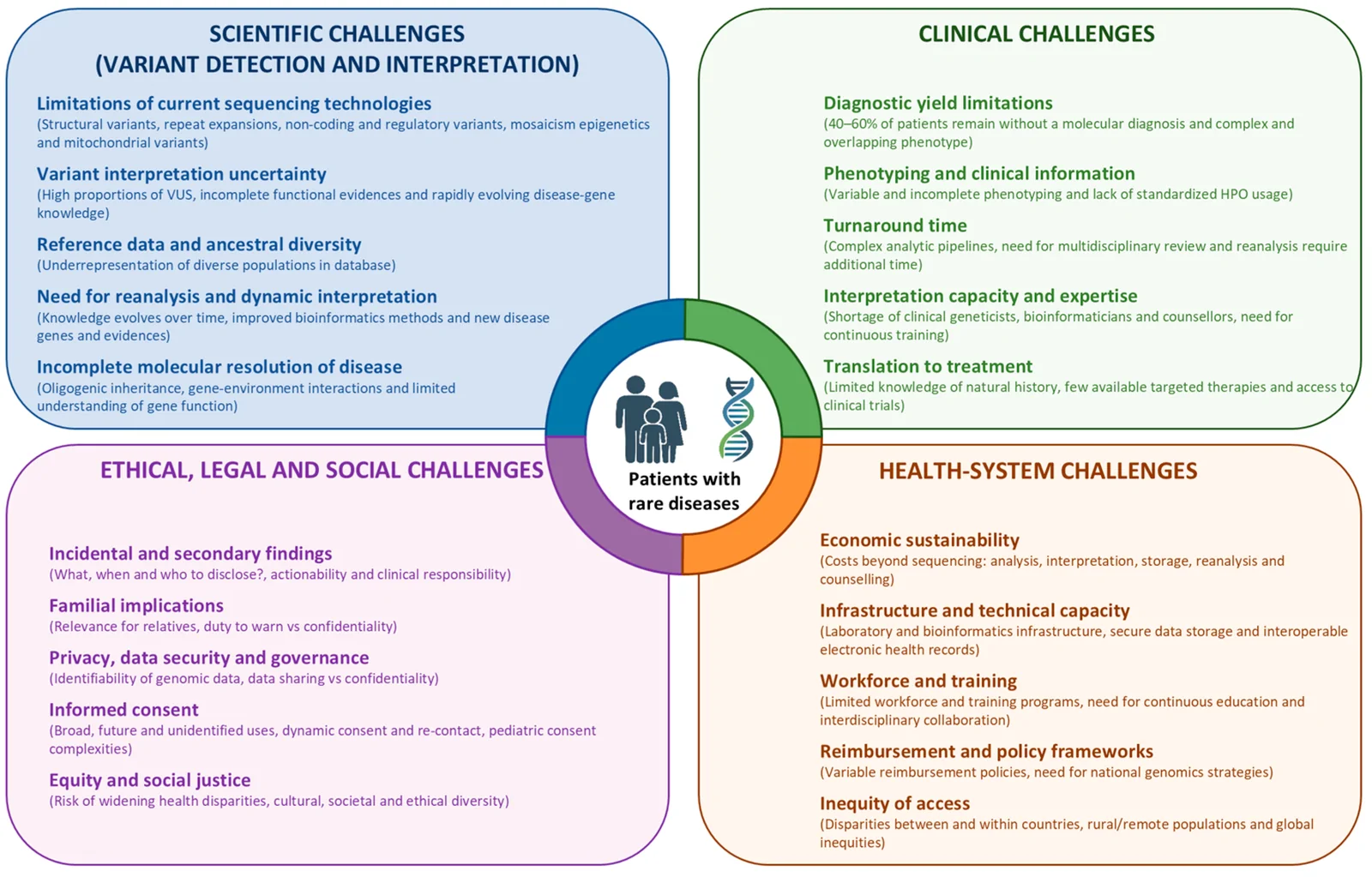
Current challenges in implementing genomic medicine for RDs. Major scientific, clinical, ethical and health-system barriers that limit the routine implementation and equitable access to genomic medicine.

While NGS, and specifically genome-scale sequencing has revolutionized the diagnosis of RD and enabled unprecedented advances in precision medicine, several scientific, clinical, ethical, and health-system challenges continue to limit its routine implementation. These barriers extend beyond sequencing technologies themselves and include difficulties in variant interpretation, ethical and legal considerations surrounding genomic information, inequitable access to testing, and the sustainable integration of genomics into healthcare systems (Figure 3). Addressing these challenges will be essential to fully realize the clinical potential of genomic medicine.

### 4.1. Challenges in Variant Detection and Interpretation

The principal challenge in RD genomics is no longer the generation of sequencing data but their accurate interpretation. Although WES and WGS have substantially improved diagnostic yield, ~40–60% of patients with suspected monogenic disorders remain without a molecular diagnosis, highlighting the complexity of genomic variation and the current limitations of diagnostic technologies [64,65,66,67].

Many pathogenic variants remain difficult to detect using conventional short-read sequencing. These include SV, repeat expansions, complex rearrangements, mobile element insertions, mitochondrial variants, deep intronic and regulatory variants, epigenetic alterations, and low-level mosaicism. Likewise, incomplete penetrance, variable expressivity, oligogenic inheritance, polygenic modifiers, and gene–environment interactions frequently complicate genotype–phenotype correlations and challenge causal inference [68,69,70].

Variant interpretation represents an equally important bottleneck. Despite the widespread adoption of the ACMG/AMP guidelines [71] for sequence variant classification, a substantial proportion of detected variants remain classified as VUS, limiting their immediate clinical utility [72]. Interpretation is further constrained by incomplete functional evidence, limited knowledge of gene function, rapidly expanding disease-gene catalogues, and the persistent underrepresentation of non-European populations in reference databases such as gnomAD. This lack of ancestral diversity contributes to disparities in diagnostic yield and increases the frequency of uncertain classifications in underrepresented populations [73].

Increasingly, periodic reanalysis of genomic data has become an integral component of genomic diagnostics. Multiple studies have demonstrated that systematic reinterpretation of sequencing data after 12–24 months can increase the diagnostic yield by approximately 5–15 percentage points (Table 1), although the magnitude varies substantially according to cohort characteristics, the interval between analyses, the breadth of the original analysis, and the extent of newly available gene–disease knowledge [27]. In some cohorts, cumulative diagnostic gains over longer follow-up are higher, particularly when reanalysis is combined with updated phenotyping and newly identified disease genes [74,75].

Emerging technologies—including LRS, transcriptomics, epigenomics, proteomics, metabolomics, single-cell approaches, and functional assays—are progressively complementing genome sequencing to improve variant detection, resolve previously unsolved cases, and provide functional evidence supporting variant pathogenicity. Together, these approaches are driving a transition from genome sequencing alone towards integrated multi-omics diagnostics.

### 4.2. Ethical, Legal and Social Implications

Genome sequencing generates information that frequently extends beyond the initial diagnostic indication. Although the terms are sometimes used interchangeably, secondary findings are deliberately sought during analysis, whereas incidental findings are detected unintentionally outside the primary diagnostic indication. The currently American College of Medical Genetics and Genomics (ACMG) recommendations specifically address the reporting of secondary findings identified in exome and genome sequencing, including medically actionable variants in genes unrelated to the primary indication, and provide an updated list of medically actionable gene–disease pairs [76,77]. For incidental findings, reporting should be guided by the clinical validity and actionability of the finding, the circumstances under which it was identified, and the consent and preferences established before testing, with appropriate clinical and genetic counselling when disclosure is considered [78].

These challenges are especially pronounced in children, where clinicians must carefully balance the child’s future autonomy against the potential benefits of early detection for both the child and at-risk family members. Decisions regarding disclosure require consideration of clinical utility, disease penetrance, age of onset, and the availability of preventive or therapeutic interventions.

Genomic information is inherently familial and uniquely identifiable, creating important challenges for privacy, confidentiality, and data governance. As international genomic data sharing becomes increasingly essential for variant interpretation and novel disease-gene discovery, robust governance frameworks are required to balance scientific collaboration with data protection and public trust [79,80]. Regulatory frameworks such as the General Data Protection Regulation (GDPR), together with standards developed by the Global Alliance for Genomics and Health (GA4GH), provide an important foundation for responsible genomic data sharing.

Obtaining informed consent has also become increasingly complex. Rather than representing a single diagnostic test, genome sequencing now constitutes a longitudinal process involving data storage, future reinterpretation, secondary analyses, research participation, and potential re-contact of patients as knowledge evolves. Consequently, dynamic consent models have been proposed to provide greater flexibility and promote ongoing patient engagement throughout the genomic care pathway.

### 4.3. Economic and Implementation Challenges

Although the cost of sequencing has fallen dramatically over the past decade, the economic challenges associated with genomic medicine extend far beyond the sequencing assay itself. Comprehensive implementation requires substantial investment in laboratory infrastructure, bioinformatics pipelines, secure data storage, multidisciplinary interpretation, confirmatory testing, periodic reanalysis, functional validation, and specialized genetic counselling services.

Significant inequalities in access to genomic medicine persist across healthcare systems owing to differences in reimbursement policies, laboratory capacity, workforce expertise, and national genomic strategies. These disparities are particularly evident between high- and low-resource settings and contribute to unequal access to molecular diagnosis, precision therapies, and genotype-driven clinical trials.

Nevertheless, a growing body of evidence demonstrates that early implementation of genome sequencing, particularly as a first-line investigation for children with suspected monogenic disorders, is increasingly cost-effective [81]. Earlier molecular diagnosis reduces unnecessary investigations, shortens the diagnostic odyssey, avoids ineffective treatments, and enables more appropriate clinical management. Future implementation efforts should therefore focus not only on reducing sequencing costs but also on demonstrating long-term clinical utility, health-economic value, and equitable integration within publicly funded healthcare systems [82,83,84,85].

## 5. Overcoming Current Barriers: Towards Equitable Genomic Medicine

Despite the remarkable advances in genome-scale sequencing, significant scientific, technical, and healthcare implementation challenges continue to limit the full clinical potential of genomic medicine. Emerging computational approaches, international collaboration, and healthcare system integration are now playing a pivotal role in overcoming these barriers and enabling more equitable access to genomic diagnosis and precision medicine.

### 5.1. Artificial Intelligence and Computational Genomics

Artificial intelligence (AI) and machine learning (ML) are rapidly transforming genomic medicine by addressing one of its greatest bottlenecks: the interpretation of increasingly complex genomic data. As sequencing technologies continue to outpace our ability to interpret the resulting variants, AI-based approaches are becoming essential for supporting clinical decision-making throughout the diagnostic workflow [86].

ML algorithms have demonstrated considerable utility across multiple stages of genomic analysis, including variant prioritization, phenotype-driven gene ranking using HPO terms, pathogenicity prediction, structural variant detection, automated literature mining, and the integration of multi-omics datasets [87,88,89]. Deep learning models are also improving the prediction of splice-altering variants, regulatory element disruption, protein structure and function, and genotype–phenotype relationships, thereby facilitating the identification of disease-causing variants that would otherwise remain undetected.

Recent advances in foundation models and large language models (LLMs) further extend the potential of AI in genomic medicine. These systems can support automated curation of scientific literature, extraction of clinically relevant evidence, generation of standardized genomic reports, and clinical decision support, substantially reducing interpretation time while improving consistency across laboratories [90,91]. In parallel, AI is facilitating the integration of genomic, transcriptomic, proteomic, metabolomic, imaging, and electronic health record data, enabling more comprehensive and personalized diagnostic approaches.

Nevertheless, AI should be regarded as a decision-support tool rather than a replacement for expert clinical interpretation. The reliability of AI models depends on the quality and diversity of the datasets used for training, and concerns remain regarding algorithmic bias, limited representation of underrepresented populations, lack of transparency, explainability, reproducibility, and regulatory oversight [92]. Prospective clinical validation, standardized benchmarking, and integration within multidisciplinary genomic workflows will therefore be essential before these technologies can be routinely adopted in clinical practice.

Collectively, AI has the potential not only to improve diagnostic yield and shorten the diagnostic odyssey for patients with RDs, but also to facilitate equitable implementation of genomic medicine by reducing analytical workload, supporting less specialized centers, and accelerating access to precision therapies and genotype-driven clinical trials.

### 5.2. International Collaboration and Data Sharing

Given the extreme rarity and marked genetic heterogeneity of most RDs, international collaboration has become indispensable for achieving accurate molecular diagnoses, establishing robust genotype–phenotype correlations, and accelerating novel disease-gene discovery. Individual institutions rarely encounter sufficient numbers of patients with the same disorder to generate meaningful clinical evidence, making secure genomic data sharing essential for both research and clinical care. Consequently, international initiatives promoting standardized data collection, interoperable infrastructures, and collaborative analyses have become fundamental pillars of genomic medicine.

Over the past decade, several global initiatives have transformed the RD landscape. The International Rare Diseases Research Consortium (IRDiRC) has established ambitious global goals to shorten the diagnostic odyssey and improve access to diagnosis and therapies for individuals living with RDs [93]. Platforms such as Matchmaker Exchange, GeneMatcher, DECIPHER, ClinVar, and the GA4GH have facilitated secure exchange of genomic and phenotypic data, enabling the identification of novel disease genes, improving variant interpretation, and promoting harmonized standards for genomic data sharing across institutions and countries [94,95,96].

The adoption of standardized phenotypic descriptors, particularly the HPO, together with the FAIR (Findable, Accessible, Interoperable and Reusable) principles for data stewardship, has further enhanced the interoperability of genomic resources and the application of AI for phenotype-driven diagnostics [97]. More recently, federated data analysis approaches have emerged as promising solutions to enable collaborative analyses while preserving data privacy and complying with national and international regulatory frameworks, including the GDPR [98].

In Europe, the European Reference Networks (ERNs) have established an unprecedented framework for cross-border multidisciplinary care by connecting expert centers for rare and complex diseases. Through virtual multidisciplinary case discussions, harmonized clinical guidelines, shared registries, and coordinated research activities, the ERNs facilitate equitable access to specialized expertise irrespective of patients’ geographical location. Building on this infrastructure, the Joint Action Integrating ERNs into National Healthcare Systems (JARDIN) aims to strengthen the integration of ERN expertise into routine healthcare, harmonize diagnostic pathways, improve interoperability between national health systems, and reduce disparities in access to genomic medicine across Europe [99]. Together, these initiatives represent a critical step towards a sustainable, collaborative, and equitable genomic healthcare ecosystem capable of supporting precision medicine for patients with RDs.

### 5.3. Towards Sustainable Implementation

The successful integration of genomic medicine into routine healthcare requires far more than continued technological innovation [100]. Sustainable implementation depends on coordinated national genomic strategies, standardized clinical guidelines, interoperable health information systems, robust laboratory infrastructure, sustainable reimbursement models, and a multidisciplinary workforce with expertise in clinical genetics, bioinformatics, and genetic counselling. Equally important is the development of governance frameworks that ensure high-quality genomic services while promoting equitable access across different healthcare settings and populations.

Several countries have already launched national genomic medicine programs to facilitate the integration of genome-scale sequencing into clinical practice, demonstrating the value of coordinated policies, centralized infrastructure, and standardized diagnostic pathways [101,102,103]. However, substantial disparities remain in access to genomic testing, specialist expertise, and reimbursement, both between and within countries. Addressing these inequalities will require harmonized quality standards, cross-border collaboration, and sustained investment in healthcare infrastructure to ensure that advances in genomic medicine benefit all patients with RDs [100,104].

Successful implementation also depends on improving genomic literacy among both healthcare professionals and patients. As genome sequencing becomes increasingly embedded in routine clinical practice, physicians across multiple specialties—not only clinical geneticists—will require training in genomic test selection, variant interpretation, communication of uncertain results, and ethical aspects of genomic medicine. Likewise, empowering patients and families through accessible genomic education and shared decision-making will be essential to maximize the clinical utility of genomic technologies and foster trust in genomic healthcare.

Finally, genomic medicine should be viewed as a continuously evolving learning healthcare system. The routine integration of genomic data with longitudinal clinical information, electronic health records, and real-world evidence will enable iterative reinterpretation of genomic findings, continuous evaluation of clinical utility and cost-effectiveness, and the rapid translation of emerging scientific knowledge into clinical practice. Such adaptive healthcare models will be fundamental for ensuring the long-term sustainability, scalability, and equitable implementation of precision medicine [105,106,107].

## 6. Conclusions and Future Directions

Genomic medicine is entering a transformative era in which WGS, particularly long-read WGS, is progressively becoming the cornerstone of RD diagnostics [41]. By enabling the comprehensive detection of single-nucleotide variants, SV, repeat expansions, complex rearrangements, mitochondrial variants, and non-coding pathogenic alterations within a single assay, LRS is poised to overcome many of the limitations of current short-read technologies. As sequencing costs continue to decline and analytical pipelines mature, lrWGS is expected to become the first-line genomic test for patients with suspected rare genetic diseases, replacing the traditional sequential diagnostic paradigm.

Future diagnostic strategies will increasingly combine genome sequencing with transcriptomics, epigenomics, proteomics, metabolomics, functional assays, and artificial intelligence to provide a more comprehensive understanding of disease biology and improve diagnostic accuracy [108]. Rather than relying on a single technology, integrated multi-omics approaches will enable more precise interpretation of genomic variation, facilitate novel disease-gene discovery, and support increasingly personalized clinical decision-making.

One of the most promising developments is the implementation of genomic newborn screening. Genome-scale sequencing has the potential to identify actionable genetic disorders before symptom onset, enabling earlier intervention, preventing irreversible disease progression, and improving long-term health outcomes [109,110]. However, widespread implementation will require robust evidence of clinical utility and cost-effectiveness, together with carefully designed ethical frameworks addressing informed consent, data governance, equity, and long-term follow-up.

The concept of longitudinal genomics is also emerging as a new paradigm in genomic medicine. Rather than representing a one-time diagnostic investigation, genomic data are increasingly viewed as a lifelong clinical resource that can be periodically reanalyzed as bioinformatic methods, disease knowledge, and therapeutic opportunities continue to evolve [111]. The integration of genomic information with electronic health records and real-world clinical data will facilitate continuous reinterpretation of genomic findings and support adaptive, learning healthcare systems.

At the same time, rapid advances in gene replacement, genome editing, RNA-based therapeutics, antisense oligonucleotides, and other precision therapies are expanding treatment opportunities for an increasing number of RDs. Consequently, obtaining an early molecular diagnosis is becoming not only the endpoint of the diagnostic journey but also the gateway to personalized treatment, genotype-driven clinical trials, and preventive care.

Ultimately, the future of RD genomics will depend not only on technological innovation but also on equitable implementation. Sustained international collaboration, responsible genomic data sharing, interoperable healthcare infrastructures, patient engagement, and global efforts to reduce disparities in access to genomic medicine will be essential to ensure that the benefits of precision medicine reach all individuals with RDs, regardless of geographic location, ancestry, or socioeconomic status. The transition from reactive diagnostics to proactive, genome-informed healthcare represents one of the greatest opportunities for precision medicine in the coming decade.

## Data Availability

The data that support the findings of this study are available from the corresponding authors upon reasonable request.

## Abbreviations

RD	Rare Disease
WES	Whole-Exome Sequencing
WGS	Whole-Genome Sequencing
NGS	Next-Generation Sequencing
LRS	Long-Read Sequencing
lrWGS	long-read Whole-Genome Sequencing
AI	Artificial Intelligence
ML	Machine Learning
VUS	Variants of Uncertain Significance
ACMG/AMP	American College of Medical Genetics and Genomics/Association for Molecular Pathology
MLPA	Multiplex Ligation-dependent Probe Amplification
CMA	Chromosome Microarray Analysis
SNP	Single Nucleotide Polymorphism
CES	Clinical exome sequencing
CNV	Copy Number Variation
SV	Structural Variants
RNA-seq	RNA sequencing
ERNs	European Reference Networks
HPO	Human Phenotype Ontology
GDPR	General Data Protection Regulation
GA4GH	Global Alliance for Genomics and Health
LLMs	Large Language Models
